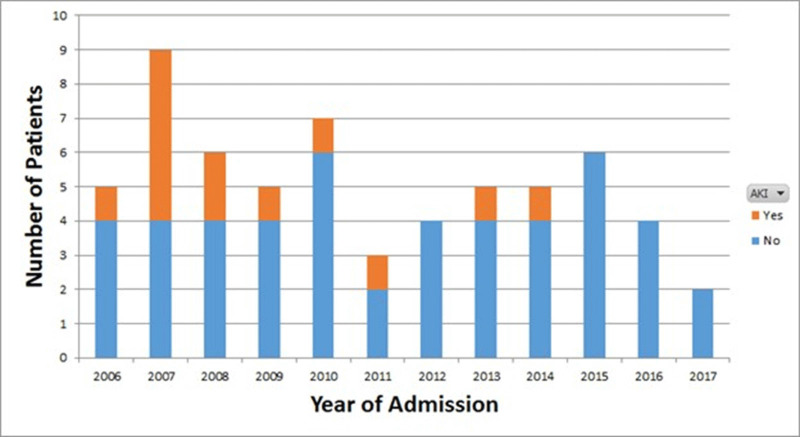# Successful Treatment of Invasive MRSA Infections in Children Using Area Under the Vancomycin Concentration-Time Curve Divided by the Minimum Inhibitory Concentration (AUC/MIC) to Measure Vancomycin Exposure

**DOI:** 10.1017/ash.2021.57

**Published:** 2021-07-29

**Authors:** Leslie Chiang, Alice Pong, John Bradley, Paige Anderson, William Murray

## Abstract

**Background:** Vancomycin is the treatment of choice for invasive methicillin-resistant *Staphylococcus aureus* (MRSA) infections. Previous guidelines issued by the Infectious Diseases Society of America (IDSA) recommended targeting vancomycin serum trough concentrations of 15–20 mg/L; however, troughs <15 mg/L are also associated with increased odds of renal toxicity. To minimize toxicity, recently updated ASHP/IDSA/PIDS vancomycin dosing guidelines recommend the use of an area under the vancomycin concentration-time curve divided by the minimum inhibitory concentration (AUC/MIC) pharmacodynamic index to measure vancomycin exposure, with an AUC/MIC ratio >400 correlating with clinical efficacy. However, data on vancomycin therapeutic drug monitoring (TDM) in children are limited. Our institutional practice since January 2009 has been to use AUC/MIC, rather than serum trough concentrations, to guide vancomycin dosing. In this study, we describe clinical outcomes in vancomycin-treated children with invasive MRSA infections using this dosing method. **Methods:** We performed a retrospective chart review of children hospitalized with invasive MRSA infections between 2006 and 2019 at Rady Children’s Hospital in San Diego, California. Clinical, microbiologic, and pharmacologic data including the site of MRSA infection, clinical failure or cure, occurrence of acute kidney injury (AKI), vancomycin MIC, vancomycin AUC, and serum trough concentrations were collected. **Results:** In total, 61 invasive MRSA cases were reviewed: 20 were admitted January 2016 through December 2008, and 41 were admitted January 2009 through June 2019 (Figure 1). Most patients did not have medical comorbidities. The most common types of infections were primary bacteremia (34%) and osteomyelitis (32%). Of 61 children, 50 (82%) had positive clinical outcomes regardless of vancomycin dosing method. Of 20 patients, 8 (40%) admitted prior to January 2009 developed AKI, compared with 5 (12%) of 41 patients admitted after January 2009. **Conclusions:** In our retrospective review, most patients had clinically successful outcomes regardless of which dosing strategy was used. We found higher rates of renal toxicity in patients who were admitted prior to 2009, with TDM based on measuring peak and trough concentrations, compared with those using AUC/MIC for TDM. Our findings suggest that AUC/MIC TDM for invasive MRSA infections may be associated with lower rates of renal toxicity.

**Funding:** No

**Disclosures:** None

**Figure 1.**